# Prospects of cognitive-motor entrainment: an interdisciplinary review

**DOI:** 10.3389/fcogn.2024.1354116

**Published:** 2024-02-21

**Authors:** Daphne G. Schmid

**Affiliations:** Department of Kinesiology, University of Georgia, Athens, GA, United States

**Keywords:** entrainment, cognitive-motor behavior, dual-task, physical activity, motor entrainment

## Abstract

Entrainment theory, a multidisciplinary concept referring to the automatic synchronization of oscillatory patterns, can be used to explain interactions across motor production, cognition, and environmental processing. This review summarizes findings from the three primary categories of naturally occurring neural entrainment: body-brain entrainment of involuntary self-produced rhythms, bottom-up entrainment between environmental stimuli and the brain, and top-down neural entrainment of self-produced processes. Studies evaluating entrainment's impact on cognition suggest that synchronized neural activation may alleviate cognitive constraints. Entrainment has also been therapeutically implemented to decrease motor production variation and enhance movement quality. When considering the evidence for entrainment's ability to decrease the attentional load of a task and increase cognitive or motor production quality, the oscillatory synchronization of a cognitive and motor task may be a promising technique that can be applied to dual-tasking. An emerging body of literature suggests that cognitive-motor entrainment may alleviate dual-task cost and, in some cases, lead to a higher quality of psychological and physiological task performance than when the same tasks are performed in isolation. We propose pathways for future research and emphasize the therapeutic relevance further experimentation on the topic of entrainment may provide. By understanding how to maximize neural entrainment's cognitive and motor benefits, scientists and practitioners may be able to harness its benefits to enhance learning and rehabilitative practices.

## 1 Introduction

Entrainment theory, a multidisciplinary concept referencing the intrinsic propensity toward oscillatory pattern synchronization, appears as a common theme in work seeking to understand interactions among an individual's bodily movement, cognitive processes, and the surrounding environment. When applied to a psychological or a physiological context, entrainment refers to the alignment among or between different types of neural activity, including cortical interneuronal communication and afferent or efferent activity between the body and the cortex. For example, an individual may consciously entrain a motor skill, such as foot tapping, with incoming sensory information, such as the beat of a song. Unconscious entrainment may also occur, where neural signals for incoming sensory information and cortical activity dictating an individual's perception synchronize. Wherever multiple oscillatory patterns are presented, the potential for entrainment is present, no matter the source of the signal.

Psychologists apply principles of neural entrainment to conceptualize patterns of attentional control (Helfrich et al., 2019). Biologists explain the regulation of involuntary physiological processes of the autonomic nervous system with this principle (Lakatos et al., 2019). Linguists use neural entrainment to explain how we perceive and interpret the meaning of words, phrases, and sentences (Ding et al., 2017), and social cognitive neuroscientists posit that the alignment of inter-brain oscillatory communication underlies consciousness and perception (Valencia and Froese, 2020). However, while the assumed mechanism behind these lines of inquiry is the same, minimal interdisciplinary integration of the various contexts of entrainment has emerged. As a consequence, the translation of results and research progression has been limited. This review addresses this void by summarizing the most prominent lines of entrainment research to promote theory development and guide future directions in cognitive-motor entrainment experimentation. By broadening the scientific community's understanding of the implications of entrainment, researchers may be able to create tasks or environments that allow individuals to capitalize on the cognitive and motor benefits it provides.

## 2 Neural entrainment

### 2.1 What is neural entrainment?

**Figure 1:**
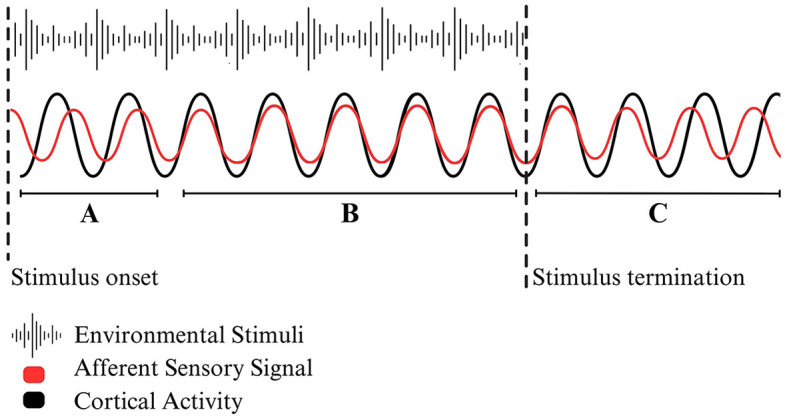
A stylized example of environmental entrainment. **(A)** Following a stimulus onset, afferent sensory signals will travel up the spinal cord toward the cortex. Depending on the timing and the intensity of the stimuli, incoming neural activity may cause a phase shift by which the frequency of cortical activity begins to align with sensory signals. **(B)** When the two oscillating wavelengths align with one another, the system has reached bottom-up environmentally driven entrainment. **(C)** When the external stimuli is terminated or changes frequency, the two systems become unsynchronized, falling out of neural entrainment.

The principle of entrainment originates from the field of physics and refers to the repetitive alignment of oscillatory patterns. Entrained processes create a stable, patterned relationship by which the peaks and troughs of independent wavelengths align (Figure 1). Notably, the repetitive, cyclical nature of oscillatory alignment distinguishes entrainment from single-phase synchronization (Bittman, 2021). When applied to neurophysiology, measurements of brain operations have led scientists to identify entrainment as a foundational element of cortical stimulation that enables the alignment of neural activity. In their comprehensive theoretical review, Lakatos et al. (2019) explain neuronal entrainment as a foundational mechanism underlying brain functioning communication that “set[s] an internal context for the modulation and interpretation of external signals or internal content based on the brain's goals and expectations.” Although research has begun to evaluate the biological markers of entrainment, the rationale behind the proposed implications of neural entrainment primarily relies on models of neural activity and theoretical explanations (Beliaeva et al., 2021).

*The Theory of Dynamic Information Selection by Entrainment* (DISE) has been used to explain neural interactions among an individual's body, perception, and environment (Lakatos et al., 2019). Proponents of DISE argue that entrained oscillatory mechanisms create a rhythmic neural context that can align external stimuli patterns with internal information processing systems. As a supramodal mechanism enhancing the quality of neural functioning, entrainment modulates sensory processing by creating fluctuating patterns of high and low cognitive excitation (Calderone et al., 2014). When neural oscillations peak, attentional capacity is magnified to absorb pertinent information, and when they are at a trough, attention decreases to filter out unnecessary information.

While neural entrainment appears to be indiscriminate regarding the brain regions in which it occurs, research suggests that the frequency of entrainment likely dictates its functionality. For example, entrainment in the delta band is thought to have the largest impact on attentional processes (Lakatos et al., 2008). In contrast, β-frequency entrainment is more closely related to motor production signaling (Guerra et al., 2016). α-frequency neural entrainment is commonly associated with working memory, perception, and consciousness, while entrainment within the theta band is most commonly linked to memory formation and hippocampal activation patterns (Clouter et al., 2017; Hanslmayr et al., 2019). Furthermore, gamma-frequency entrainment is thought to influence attention and coordination of cognitive processing.

The entrainment of one neural frequency rarely occurs in isolation. When cross-frequency coupling occurs, low-frequency neural oscillations are thought to modulate high-frequency neural oscillations (Obleser and Kayser, 2019). Lakatos et al. (2008) demonstrate how gamma waves, which are also associated with attention and cortical processing, are influenced by delta band rhythmicity to produce synchronization across frequencies. Furthermore, cross-synchrony of neural frequencies contributes to working memory formation, perception, consciousness, and language comprehension (Palva and Palva, 2007; Obleser and Kayser, 2019). To explain frequency modulation and entrainment interactions, Fries (2005) proposed the communication-through coherence (CTC) hypothesis, which suggests that phase-locking of neural activation patterns may be the mechanistic foundation for neural communication. Rhythmic activation patterns at different frequencies are thought to contribute to the formation of selective pathways for cortical communication, promote cognitive flexibility, and prevent errors that may be caused by non-coherent activations or non-neural oscillations. Experimental evidence suggests that “gamma and beta rhythms modulate input gain, and their coherence subserves effective connectivity” (Fries, 2015). When principles from the CTC hypothesis and the DISE theory are conflated, the principle of entrainment can be applied to various settings to explain neural, behavioral, and motor functioning.

The neural substrates underlying entrainment are relatively unknown. EEG analyses evaluating the presence of entrainment throughout the brain suggest that phase-synchronization of neural activation patterns occurs indiscriminately across the cortex (Lakatos et al., 2019), but the mechanistic underpinnings of entrainment are less understood. Some researchers have begun to address this gap in the research by identifying relationships between neural entrainment and cognitive outcomes. For example, delta-frequency entrainment within the thalamocortical circuit has been linked to auditory processing (Barczak et al., 2018), and theta wave entrainment within the hippocampus and the medial temporal lobe is associated with memory encoding and retrieval (Hanslmayr et al., 2019; Köster et al., 2019). However, further research is needed to clarify these relationships to reveal the underlying mechanisms and neuronal substrates behind neural entrainment.

### 2.2 Neural entrainment and psychological theory

When addressing the behavioral impact of entrainment, DISE predicts that stimuli synchronization enhances cognitive functioning through temporally concurrent processes that facilitate one's attention to and perception of stimuli (Haegens and Zion Golumbic, 2018). Whereas neurophysiological measurements provide helpful insight into the neurobiological activation patterns underlying cortical activity, psychological theories and models help scientists conceptualize the behavioral outcomes associated with biological changes through abstract descriptions of cognitive functioning (Kay, 2018). The following section briefly discusses entrainment theory in relation to prominent psychological theories of attention and information processing.

Attention is assumed to be limited and selective. When interpreting attention through the *Theory of Selection*, an individual is thought to direct their attentional orientation through voluntary choices using “top-down” processing or involuntary choices using “bottom-up processing” (Schmidt et al., 2018). Both options require the concurrent inhibition of attention to an alternative factor to increase the allocation of attention to one stimulus. After information passes through the attentional filter, it must be organized and acted upon. Because attention constrains stimuli processing, one's perception will change when a situation requires attentional reallocation. To explain this procedure, *Information Processing Theory* uses a computational metaphor that compares the human brain's ability to take in, process, and respond to information to a computer system. The three main stages contributing to information processing are stimulus-identification, response-selection, and response-execution. Stimulus identification relies on the sensory system to detect environmental stimuli and send afferent signals. The response-selection phase requires the individual to determine how to respond to a detected stimulus, and the reaction is translated into a neural command in the response-programming stage.

Because neural activity is viewed as a limited capacity resource, the foundational concepts of attention and information processing have been integrated into broader theories that explain how cortical processes may interact and influence cognitive output as a whole. *Cognitive Energetics Theory* proposes that all cortical activity is limited by regulatory costs of neural activation that force attentional processes to divide and allocate the arousal, effort, and activation of resources required to perform a task (Sanders, 1983). *Cognitive Load Theory* extends this notion by explaining how a fixed processing capacity also bounds the resultant information that passes through attentional filters. Therefore, this cortical activity capacity rate limits the brain's ability to identify a stimulus and respond through the information processing pathway. But what if there was a way to expand functioning capacity?

**Figure 2:**
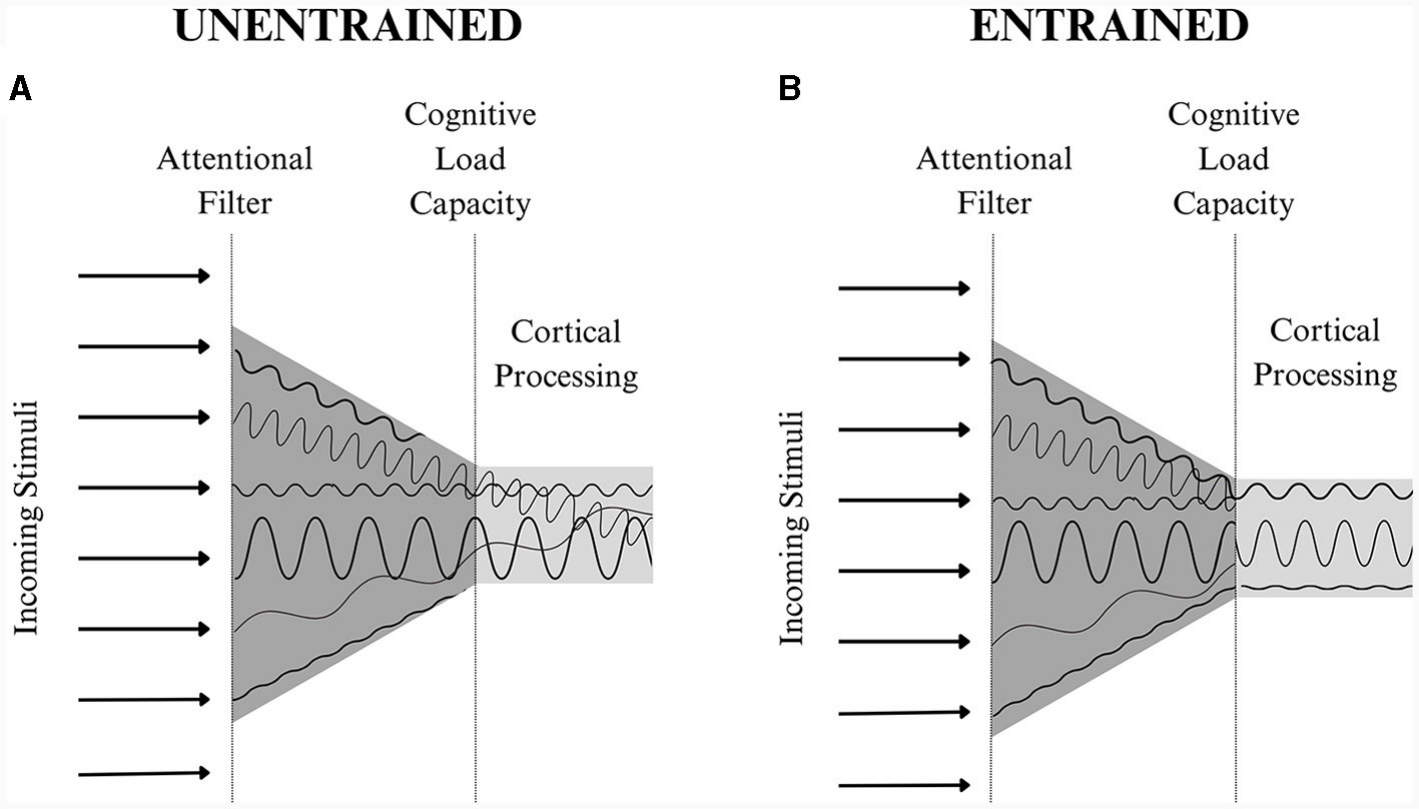
A theoretical representation of neural entrainment's effects on cognitive processing. The arrows on the left of each diagram represent different sources of stimuli. According to the Theory of Selection, an individual's goals and past experiences will dictate what information passes through the attentional filter. This information is then organized and transmitted to the appropriate areas in the cortex. The unique qualities of each stimulus create a neural wavelength with a distinct frequency and amplitude shown at the center of each diagram. In **(A)**, the conflicting neural signals create noise, increasing cognitive load. In this instance, a cortical processing capacity, detailed in Cognitive Load Theory, would prevent the transmission of all of the stimuli being attended to and limit cognitive functioning. However, in **(B)** the entrainment of multiple sources of stimuli increases one's attentional capacity as the noise across the entrained wavelengths is minimized. This enhances the volume of information one can attend to, transmit to the cortex, and process through cognitive functions. Ultimately, neural entrainment allows an individual to maximize cognitive processing, enhancing their ability to perceive and respond to information from their body and environment.

When considering neural entrainment's implications within the context of the theories above, aligning neural oscillations would likely minimize noise across cortical signals and create patterns of expected activation, decreasing neural load. In fact, multiple cognitive processing theories propose psychological principles that complement the neurobiological implications of entrainment theory. *The Dynamic Attending Theory* (Jones, 1976; Phillips-Silver et al., 2010; Bauer et al., 2015), which states that sequences presented in a predictable pattern facilitate stimuli processing, and the *Neural Resonance Theory* (Large and Snyder, 2009), which explains how the brain synchronizes processing of non-periodic stimuli, are often used to explain neural entrainment's effects. These theories suggest that rhythmic, anticipated events will be more efficiently encoded due to enhanced perceptual sensitivity (Hanslmayr et al., 2019). Within the context of neural entrainment, both theories would support the notion that by aligning neural activation patterns, the cognitive effort required to carry out the entrained functions decreases, freeing up more cortical space, magnifying the quality of cortical output (see Figure 2 for more information).

While there are research findings supporting the causal propositions posed by these theories, their mechanistic foundations await determination. In their critical review of entrainment's impact on sensory processing, Haegens and Zion Golumbic (2018) cautioned readers of methodological flaws in entrainment studies and emphasized the need to assess the relationship between neural entrainment and perception. By breaking down entrainment's driving factors, the following sections will summarize the current understanding of its biological and neurological foundations and propose ways to integrate concepts and methodological principles from different research fields exploring the concepts of neural entrainment.

### 2.3 Categories of neural entrainment

**Figure 3:**
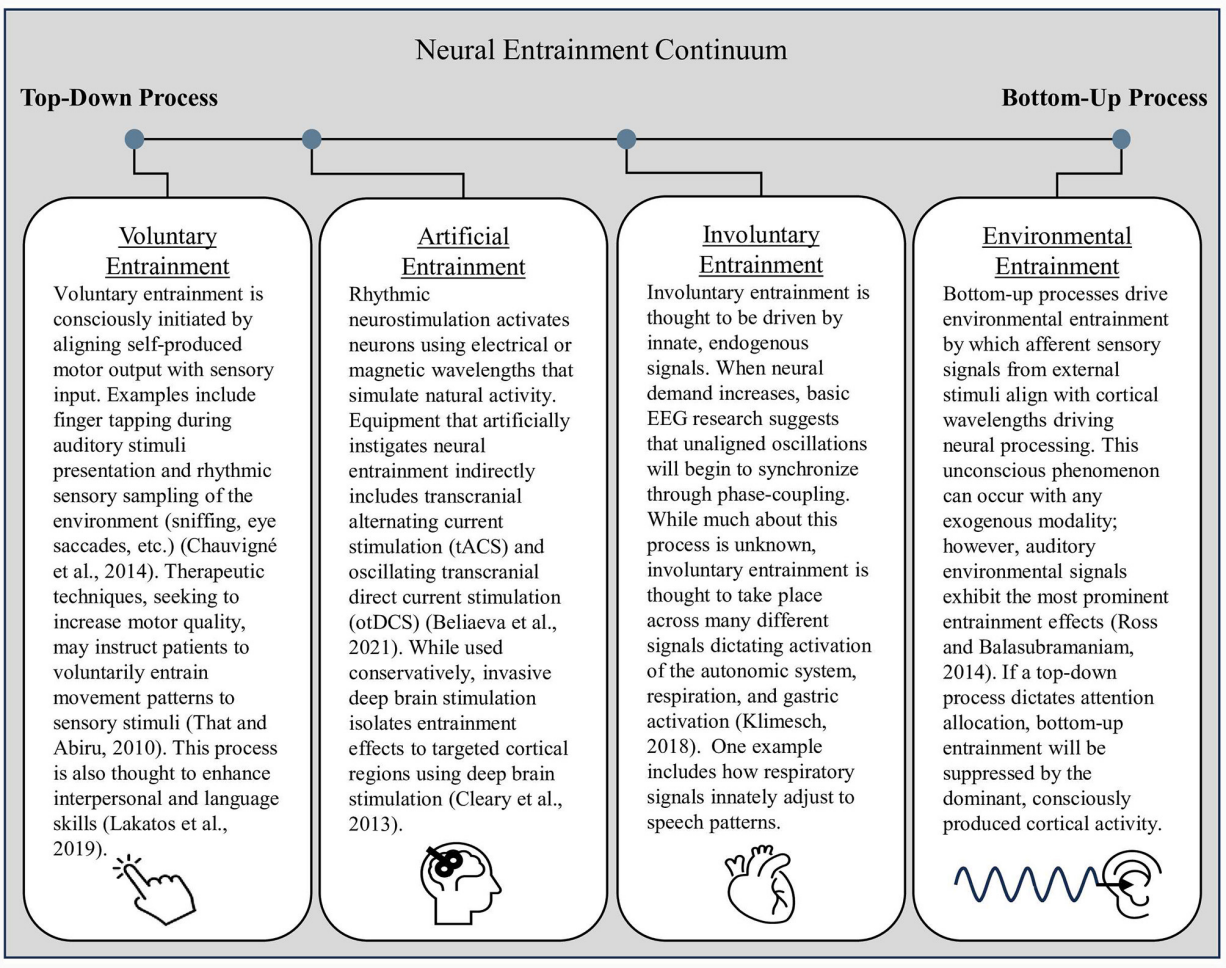
The neural entrainment continuum. Top-down entrainment processes influence cortical activation patterns that alter attentional orientation and efferent signaling. In contrast, bottom-up entrainment, represented on the far right of the continuum, is driven by afferent neural signaling that travels up to the cortex, influencing cortical activation patterns.

Neurophysiological evidence points toward three primary categories that cause neural entrainment: body-brain entrainment of involuntary or voluntary self-produced rhythms and neural activity, artificially induced neural entrainment from non-invasive stimulation, and entrainment between environmental stimuli and the brain (Lakatos et al., 2019). The former are top-down processes in which neural activation patterns and cognitive perceptions drive entrainment effects, while the latter category is a bottom-up process in which external, environmental factors influence cognitive and motor output. Because of similarities across the behavioral and cognitive factors involved in different forms of entrainment, Figure 3 organizes each type along a continuum based on whether their driving factors rely on top-down or bottom-up processes. For readers who would like an in-depth explanation of entrainment differentiation, Lakatos et al. (2019) provide a helpful review of the mechanisms behind and roles of neuronal entrainment.

#### 2.3.1 Self-produced entrainment

Self-produced entrainment uses naturally occurring physiological activation patterns to align cortical oscillations. The innate process relies on endogenous activity to initiate entrainment. One's intention or lack thereof to instigate self-produced entrainment dictates the process's classification as voluntary or involuntary.

*Voluntary:* With the ability to supersede other forms of naturally occurring entrainment, voluntary self-produced entrainment describes a top-down process by which the conscious initiation of a motor process influences the rate of sensory sampling and perceptual processing. Studies using EEG analysis show how efferent neuromotor signal oscillations entrain with afferent sensory signals. This process typically occurs through rhythmic environmental sampling, which is thought to enhance intracortical communication and decrease neural demand by creating patterns of attentional focus that benefit perceptual ability. For instance, producing visual saccades or sniffing patterns allows the brain to anticipate sensory input and allocate attention to incoming stimuli in a synchronized pattern (Schroeder et al., 2010). Other examples include how fine and gross patterned limb movement contributes to the perception of auditory stimuli (Falk and Dalla Bella, 2016) or how distinct rhythmic speech patterns influence one's understanding of language and verbal communication with others (Zoefel, 2018). Ultimately, self-produced voluntary entrainment allows individuals to optimize goal-directed neural activity through conscious prioritization, allowing it to supersede all other involuntary, naturally occurring entrainment patterns discussed below.

*Involuntary:* Involuntary self-produced entrainment primarily occurs through interactions between cortical activity and autonomic nervous system activation patterns that drive biological rhythms, including heart rate regulation, respiration patterns, and the glycolytic cycle (Jiménez et al., 2022). Some researchers propose that this type of entrainment likely provides an evolutionary advantage by decreasing noise across interoceptive processing, magnifying the brain's available resources for cortical activity (Buzsáki et al., 2013). As the body and the brain constantly work to maintain vital physiological processes, neural activation patterns are thought to align and interact to maintain homeostasis and benefit cognitive functioning (Boyadzhieva and Kayhan, 2021). To explore this concept, a study by Garfinkel et al. (2013) visually presented word lists in alignment with real-time electrocardiograph (ECG) heart rate readings, producing an induced state of involuntary entrainment. The authors report that memory for words was better when stimuli were presented in line with diastole and worse when presented during systole. These findings suggest autonomic signal entrainment with cortical oscillations may influence cognitive functioning due to entrainment's modulation of attentional processes (Garfinkel et al., 2013). Nevertheless, more research is needed to clarify this relationship, as little is understood about the mechanisms that drive entrainment within the body and between the body and the brain.

In summary, self-produced forms of entrainment are driven by internal signaling, whether consciously produced through voluntary entrainment or driven by subconscious innate processes as involuntary entrainment. Voluntary entrainment relies on top-down signaling initiated by prefrontal cortex activity. In contrast, involuntary entrainment lies in the middle of the continuum between top-down and bottom-up processing. Please see Figure 3 for further delineations regarding entrainment categories across the top-down–bottom-up entrainment continuum.

#### 2.3.2 Artificially induced entrainment

Top-down neural entrainment can also be induced using neuromodulatory techniques. Invasive and noninvasive neural therapies influence cortical activation patterns through the artificial delivery of electromagnetic stimuli that trigger neural activity. Transcranial magnetic stimulation (TMS) and transcranial alternating current stimulation (tACS) are most frequently used to produce entrainment synthetically by synchronizing natural neural activity with an oscillating input stream of magnetic or electrical currents. These non-invasive entrainment techniques have enhanced perceptual ability, working memory, and motor production quality (Thut et al., 2011; Helfrich et al., 2014). Neural entrainment may also be artificially induced using deep-brain stimulation to more accurately target specific cortex regions. Using this invasive technique, deep brain stimulation has been shown to entrain neural activity at the globus pallidus, disrupting atypical neural activity caused by Parkinson's disease (Cleary et al., 2013) and at the medial septum as a treatment for temporal lobe epilepsy (Cole et al., 2022). However, some studies evaluating the effects of invasive deep-brain stimulation on entrainment and cognition report contradictory effects. When cortical areas associated with memory are stimulated at a frequency that leads to neural entrainment, negative (Kim et al., 2018) and positive (Inman et al., 2018) cognitive effects have been reported.

Both invasive and non-invasive artificially induced neural entrainment are promising techniques that may alleviate some neurological conditions and influence cognition. It is important to note that most studies evaluating the effects of artificially produced entrainment use a small sample size. Replication studies using larger sample sizes are needed to help clarify how different neural stimulation techniques may vary in their ability to induce entrainment and influence cognitive functioning successfully (Hanslmayr et al., 2019).

#### 2.3.3 Environmentally produced entrainment

When entrainment is environmentally produced, afferent neural activity from sensory stimuli aligns with intracortical wavelengths. Research findings suggest that this bottom-up classification of neural entrainment is a natural, involuntary process. To achieve environmental entrainment, the incoming oscillatory stimulus produces systematic patterns of neural information that align with pre-existing neural activity. The stimuli must originate from an external source and cannot be generated through individual motor production, such as auditory information from self-generated speech or tactile information from finger tapping. Because of the brain's limited capacity for resources, specifically attention, the incoming stimuli that present the strongest will supersede any other incoming oscillatory information and be the strongest candidate to produce environmentally-driven entrainment (Calderone et al., 2014). While bottom-up entrainment is automatic, it can be superseded by top-down, voluntary entrained signals. This permits individuals to suppress incoming stimuli and produce a desired outcome (Lakatos et al., 2019).

Numerous studies report how neural entrainment of repetitive and predictable sensory stimulation is related to enhanced cognitive functioning (Schmidt-Kassow and Kotz, 2008; Schmidt-Kassow et al., 2009, 2013a; Gu et al., 2015; Hanslmayr et al., 2019; Lakatos et al., 2019). Researchers commonly alter the timing of stimuli presentation to systematically evaluate the effects of environmental entrainment. Results tend to be relatively stable, with the predictable, entrained presentation of stimuli facilitating memory (Brochard et al., 2013) and attentional processes (Bolger et al., 2013). Entrainment of neural oscillations during cognitive tasks can be found in all electroencephalography (EEG) bands, with common targets at P300 event-related potentials (ERPs) (Schmidt-Kassow et al., 2009), P600 ERPs (Schmidt-Kassow and Kotz, 2008; Canette et al., 2020), and FN400 ERPs (Garcia-Argibay et al., 2019), which are associated with selective attention and stimuli processing, language processing. Furthermore, bottom-up sensory entrainment studies report positive impacts on memory processes in particular (Benchenane et al., 2011). For example, experiments presenting visual stimuli rhythmically (Jones and Ward, 2019) or paired with a cyclic light flicker paradigm (Williams, 2001) report enhanced memory processing and the presence of neural entrainment through EEG analyses. The rhythmicity and predictability of stimuli presentation are thought to influence neural processing and provide mnemonic effects by creating windows of enhanced attention that facilitate item encoding.

In summary, each category of neural entrainment results in the alignment of oscillating wavelengths. Though the resultant entrainment may be the same, self-produced, artificially induced, or environmentally produced entrainment categories are distinguished by the phase shift's driving factor. For example, an individual may produce voluntary entrainment by matching their steps to the beat of a song. However, the same result could unconsciously be produced through the influence of environmental entrainment (please see Repp and Su, 2013 for a helpful review of sensorimotor primary studies on this topic). EEG findings suggest that entrainment's cognitive benefits regarding the frequency and location of cortical activation are indiscriminate, making it a versatile technique that can be used to enhance desired cortical activity and suppress atypical neural functioning. Research across disciplines points toward the importance of oscillatory activity for physiological functioning and the benefits of neural entrainment in various applications. While promising, there are inconsistencies across this body of research (Haegens and Zion Golumbic, 2018). More research is needed to clarify the mechanisms behind entrainment and to understand the intricacies of its cognitive effects.

## 3 Clinical applications of neural entrainment

Neural entrainment is thought to maximize cognitive processing potential and decrease cortical activity strain (Calderone et al., 2014). In the 1990s, rehabilitative programs designed to enhance the quality of movement production began to popularize, applying entrainment principles to therapeutic techniques. Because entrainment of neural activity is thought to increase the efficiency and efficacy of neural communication, researchers have suggested that oscillatory rhythms may synchronize with repetitive neural activity, producing stable motor activation patterns (Chauvigné et al., 2014). Auditory and visual stimuli are most frequently used to instigate environmentally-driven entrainment in rehabilitative practices. Because the influence of these stimuli is driven by bottom-up processes, this allows the researcher or therapist to make slight adjustments to the stimuli presentation, maximizing the potential for beneficial effects.

### 3.1 Entrainment therapy

Research evaluating rhythmic auditory stimuli is particularly popular in neuromotor rehabilitation techniques that draw from principles found in neurological music therapy (Thaut, 2013). Neurobiological foundations lie in auditory projections within the cerebellum and audio-motor pathways in the cortex and reticulospinal tract of the spinal column (Thaut and Abiru, 2010). The proximity of cortical auditory-motor pathways facilitates the synchronization of neural activation patterns across these brain regions and increases the likelihood of neural entrainment between oscillations from afferent auditory signals and efferent motor signals. Entrainment is driven by the period, or the interval between two beats, of rhythmic auditory signals (Thaut et al., 2014). While many individuals will unconsciously undergo bottom-up entrainment by which their motor signals align with auditory oscillations, many people require cuing to instigate top-down entrainment by which the individual seeks to match their motor activation periods to the oscillations of auditory periods from external stimuli (Moens et al., 2014; Dotov et al., 2019). Because a rhythmic sound requires consistent periodization, this type of auditory-motor entrainment is thought to stabilize efferent motor signals that produce a cyclical movement pattern. A variety of motor production patterns have been shown to be influenced by auditory cuing, including walking and running gait, and hand functioning (Thaut and Abiru, 2010; Thaut et al., 2014; Buhmann et al., 2018).

Understanding the relationship between neural activation, auditory stimulation, and motor production facilitates the use of rhythmic auditory stimulation as a rehabilitative tool. This approach seeks to synchronize motor movements with rhythmic stimuli to decrease motor production variation and alleviate neuromotor impairments. A common way to test this approach is to use a paradigm in which participants tap their fingers to an auditory rhythm. When applied, individuals tend to be able to cue motor functions more efficiently, as reflected through changes in neural activation patterns measured with EEG and electromyography (Thaut and Abiru, 2010). Similar results are also frequently seen during auditory-motor coupling of gait patterns in a healthy, young adult population through measurements of relative stepping phase angle, resultant stepping vector length, stepping asynchrony, and tempo matching accuracy (Moumdjian et al., 2018).

Additional research has shown that the mechanistic benefits of cognitive-motor entrainment translate to real-world settings to enhance and optimize walking and running performance in individuals with different pathologies (Buhmann et al., 2018). Examples of the successful implementation of rhythmic auditory stimulation include reports of increased gait quality in children with cerebral palsy, older adults following a cerebrovascular accident, and adults with Parkinson's disease (McIntosh et al., 1997; Thaut and Abiru, 2010; Ghai et al., 2018). While benefits from auditory-motor entrainment therapy are consistently found, individual differences may impact the magnitude of its effect. Factors that may influence this relationship include gender (Buhmann et al., 2018), unique neurophysiological diagnoses (Schaefer, 2014), age, and stage of sensorimotor development (Thaut and Abiru, 2010). Ongoing research seeks to further clarify which individuals may have the highest propensity to benefit from auditory-motor entrainment therapy and understand its most appropriate applications. Along with the clinical strategies discussed above, which rely on self-produced voluntary entrainment and bottom-up, environmental entrainment, artificially induced entrainment also shows promise in therapeutic application.

### 3.2 Artificial entrainment and motor production

Artificially induced entrainment techniques typically activate the cortex through electrical or magnetic stimulation. Research suggests that neural entrainment is instigated when artificial electrical signals stabilize atypical patterns of neuronal firing (Cleary et al., 2013; Sermon et al., 2023). The stable pattern of neural activity within a specific brain region encourages other sources of neural activity to entrain to the emitted wavelength's frequency and amplitude. As many movement disorders are instigated by atypical neural activation of alpha motor neurons, neural entrainment therapies seek to decrease the coefficient of variation across motor signal activation. In individuals with Parkinson's disease, deep-brain stimulation may be used to enhance neuromodulation. This therapy has effectively diminished pathological tremours when applied at the subthalamic nucleus and the globus pallidus internus (Rodriguez-Oroz et al., 2005; Fischer et al., 2020). Furthermore, tACS entrainment has been shown to help individuals manage motor control output by stabilizing neural oscillation patterns disrupted by neurodegenerative diseases or cerebrovascular or cardiopulmonary events (Takeuchi and Izumi, 2021). Experimental applications of tACS-induced entrainment also report enhancements in motor functioning following stroke (Hsu et al., 2012) and alleviate cognitive deficits associated with dementia (Elyamany et al., 2021).

Using non-invasive brain stimulation to induce entrainment artificially may also accelerate the development of motor learning and skill development. Researchers have begun to explore how augmented neural stimulation may influence the physiological and cognitive aspects of motor production. By applying sinusoidal electrical stimulation in targeted brain areas, tACS is thought to produce entrained patterns of neural activation that magnify the rate of motor learning (Colzato et al., 2017). Following the rationale used to describe entrainment's effect on psychological outcomes, the synchronization of neural activity creates fluctuating patterns of attentional capacity, facilitating learning. Experimental findings report that tACS may decrease interference effects and stabilize a motor learning task (Pollok et al., 2015). It is important to note that while studies report tACS's facilitation of motor learning, there is little evidence to show that entrainment is the true mechanism of action behind these results. Additionally, positive outcomes have been reported in studies completed in controlled environments with small sample sizes. As of now, there is little evidence to support that these findings will translate to alternative environments or lead to alterations in sports or physical activity performance in a competitive environment.

Artificially induced entrainment introduces a unique perspective when compared to alternative forms of entrainment. Although procedural restrictions limit its application in many real-world settings, experimentation using this form of entrainment provides insight into the techniques by which entrainment's effects and therapeutic potential may be maximized. Because artificial entrainment allows researchers to target distinct regions in the brain, this methodology can be used to parse out whether or not specific brain regions may differ in their ability to benefit from neural entrainment. Further exploration on this topic may allow clinicians to determine which pathologies benefit most from entrainment therapy and learn which application techniques hold the most promise of positive effects.

## 4 Dual-task cognitive-motor entrainment

As phase synchronization of neurobiological activation patterns may benefit both motor and cognitive functions separately, entrainment research emerged to explore the effects on cognitive-motor dual-task performance. The main questions being: Would cognitive-motor entrainment influence the quality of cognition, motor production, or both? and Would these effects result in positive or negative changes? Because dual tasks require concurrent allocation of attentional processes, they are often associated with a decline in the production quality of one or both tasks. However, when considering the evidence for entrainment's ability to decrease the attentional load of a task and increase cognitive or motor production quality, the oscillatory synchronization of a cognitive and motor task may alleviate the traditional costs of dual-task performance.

Traditionally, when cognitive and motor tasks are performed in tandem, adults often experience null or adverse effects on cognitive output (Loprinzi et al., 2019a,b) and a possible decline in the quality of motor production as well (Plummer, 2009). Because of the brain's finite attention and cortical processing capacities, simultaneously performing two tasks requires prioritizing and selecting tasks to direct attention toward. However, when the secondary task is not allocated the same cognitive resources as it would have received in a single-task scenario, this often leads to a decline in the secondary task's performance quality. In their review of the neural correlates associated with cognitive-motor dual-tasking, Leone et al. (2017) report that dual-task conditions often instigate cortical activity in areas of the brain that were not associated with activity during either single-task performance. The increased cognitive load resulting from dual-task interference is often observed across cortical areas associated with information processing and motor control. These findings suggest that the decline in behavioral performance often seen as a result of cognitive-motor dual-tasking is likely due to increased cortical load and strained cognitive resources.

The neurobiological evidence, which indicates that increased cortical effort is required to perform a cognitive-motor dual-task, supports conclusions presented previously concerning theoretical explanations of cortical processing (i.e., the theory of selection and cognitive load theory). Furthermore, the predictions derived from these theories suggest that neural entrainment may potentially alleviate a portion of dual-task cost. While a task's intensity or difficulty may influence the magnitude of change instigated by entrainment's effects, neural entrainment's ability to decrease a task's cognitive load provides a helpful strategy by which individuals may be able to overcome dual-task interference. A recent review of cognitive-motor interference noted that experiments implementing entrainment methodologies tend to contradict the norm of reporting dual-task performance detriments and instead have found mnemonic or beneficial cognitive effects (Tomporowski and Qazi, 2020). Through systematic experimentation evaluating the influence of cognitive-motor entrainment on task performance, these studies indicate promising behavioral and neurological effects. For example, Schmidt-Kassow et al. (2010) asked young adults who were low-span readers to encode French-German word pairs in tri-weekly training sessions across 3 weeks. During the training sessions, participants cycled to a rhythmic, auditory beat or sat stationary on an ergometer as auditory stimuli were presented consistently. At the end of each week, participants completed a cued recall test. For individuals in the cycling condition, word presentation was entrained to the participant's rate of cycling. While groups acquired the words at a similar rate across weeks, the spinning groups always produced higher free recall memory scores at every testing session. ERP results from separate EEG analyses of a very similar task show that participants in the spinning group had larger N400 peaks that were synchronous with the stimuli presentation. As N400 peaks are associated with encoding and processing semantic information, researchers concluded that synchronizing oscillatory patterns from cognitive and motor sources leads to neural activity entrainment and enhanced memory performance. In a similar study (Schmidt-Kassow et al., 2014), the same research group evaluated the effects of cognitive-motor entrainment on a different physical activity modality, treadmill walking. Participants encoded word pairs, whose presentation was entrained to individual stepping patterns. In the cognitive-motor entrainment condition, participants remembered more words encoded during cognitive-motor entrained treadmill walking than during sedentary sessions.

To understand the underlying modalities of these findings, EEG analyses can also be used to depict how auditory-motor entrainment facilitates attention through neural synchronization. This method has also been tested across a series of studies in which auditory-motor entrainment was hypothesized to narrow attentional processes by creating rhythmic peaks of high and low attentional states. In each study, participants completed an auditory oddball paradigm in which they were asked to respond to incongruent stimuli (Schmidt-Kassow et al., 2013b, 2019, 2023). Participants concurrently cycled to the rhythm of the stimuli presentation or sat stationary. EEG data consistently supported the hypotheses as entrained neural activation was seen in larger P300 ERPs (Schmidt-Kassow et al., 2013a) and pre-stimulus beta-power (Schmidt-Kassow et al., 2023). Moreover, these findings were unique to active synchronization processes and not found in paradigms where participants may have experienced passive Entrainment (Conradi et al., 2016). The researchers suggest these neural changes may contribute to enhanced cognition, particularly memory performance when individuals are presented with a similarly designed auditory-motor paradigm (Schmidt-Kassow et al., 2019).

While entrainment's influence on motor movement quality is less studied compared to its behavioral effects, entrainment may also alleviate increased motor movement variability often associated with dual-task paradigms. Traditional dual-task consequences are often seen in gait destabilization, as revealed through higher CoV measures of swing time (Nankar et al., 2017), stride length (Agostini et al., 2015), and most commonly, through decreases in overall walking speed (Beauchet et al., 2005; Bayot et al., 2018). However, studies evaluating entrained cognitive-motor dual tasks report inverse results. In an experiment by Schmidt-Kassow et al. (2013b), participants were asked to learn 80 Polish-German vocabulary pairs while sedentary, while cycling, or before a low-intensity cycling session. Those who entrained their cycling pace to auditory stimuli presentation had smaller coefficients of pace variation when compared to traditional dual-task performance *and* retained more word pairs during long-term memory assessments. This experiment suggests that neural entrainment may concomitantly enhance the performance of two separate tasks. However, the small number of experiments on this topic limits the conclusions that can be drawn from their findings. The following section will provide recommendations for further experimentation on the impact of cognitive-motor dual-task entrainment and methodological suggestions to maintain experimental integrity.

### 4.1 Questions and future directions in cognitive-motor entrainment

The current data on cognitive-motor dual-tasking employs many methodologies to explore research questions, resulting in ambiguous conclusions about how conflicting exercise parameters may lead to differential task performance outcomes. In prior work evaluating cognitive-motor entrainment, Schmidt-Kassow and Kaiser (2023) emphasize this point by stating,

“Future studies should try to clarify whether synchronization was actually the mechanism that led to a narrowed attentional focus, or which other parameters (restricted vs. free motor activity, exercise intensity, motor modality, cognitive processes under investigation) may have contributed to the combined effects. (p. 13)”

The psychological theories discussed previously (i.e., Information Processing Theory, Cognitive Load Theory) predict that physical activities with high attentional demands would deplete neural resources that could be applied to cognitive processes due to limited attentional capacities. Therefore, high-intensity, multi-limb, coordinative, or lengthy physical activities may prevent an individual from reaping potential cognitive benefits. Other factors to consider could include free (overground) movements in heterogeneous environments vs. controlled movements in homogenous environments (such as riding a cycling or rowing ergometer, etc.) and indoor vs. outdoor activities. Most cognitive-motor dual-task reviews report that physical activities with fewer degrees of freedom are more likely to lead to positive benefits (Schmidt-Kassow and Kaiser, 2023). Following these recommendations, low-moderate intensity is recommended over high-intensity physical activity. Indoor-controlled activities are preferred over outdoor, highly-variable activities, such as overground running, and simple, single-limb activities, such as a cycle ergometer, would be preferred over activities requiring limb coordination or postural control, such as running or wall climbing (Lambourne and Tomporowski, 2010; Tomporowski and Qazi, 2020). Prominent researchers have called for further systematic research to clarify these intricacies in the relationship between physical activity and cognition (Lambourne and Tomporowski, 2010; Loprinzi et al., 2018). The same concern should be considered when evaluating cognitive motor entrainment. While research indicates that synchronizing behavioral and motor functions will minimize the amount of neural resources used, alternative factors that increase motor complexity may cancel out or override potential benefits. The methodological design of future studies should consider these factors when determining the type and volume of physical activity that will be incorporated into an entrainment experiment.

The direction and prioritization of entrainment's effects on task outcomes are also unknown. Perhaps neural entrainment's effects on cognition and motor production occur at the same magnitude, or one task type may tend to benefit more than another. In their review of research evaluating the cognitive effects of consecutive motor activity, Schmidt-Kassow and Kaiser (2023) discuss how the specific cognitive tasks and motor modality are also likely to interact. Divergent cognitive processes tend to benefit more from unstructured, free activity while convergent processes benefit from physical activity that requires minimal attention for execution. Systematic experimentation evaluating different cognitive and motor variables is needed to clarify if there is an interaction between these variables and, if so, what specific properties of entrained cognitive-motor tasks have the greatest potential for success. For example, the principle of entrainment could be applied to an embodied cognition intervention where children play a physical activity game that synchronizes information presentation with stepping, hopping, or jumping (Mavilidi et al., 2021). However, the current research on entrainment does not provide helpful guidelines by which this intervention should be designed. If the ultimate goal of this research is to understand how we can most effectively use entrainment in a real-world setting, understanding if a cognitive or behavioral task will be prioritized over another and how the qualities of other tasks may mediate and treatment effects will help researchers identify scenarios in which neural entrainment has the largest potential for positive outcomes.

Further research clarifying the mechanisms behind entrainment and its neural substrates may also help researchers identify strategies to maximize interventions hoping to harness its cognitive and motor benefits. Techniques that artificially induce entrainment, such as TMS, tACS, or deep brain stimulation, target distinct brain regions and neural networks. Experimentation that compares the efficacy of entrainment's effects within different neural regions and across different cognitive processes or motor outputs would help researchers distinguish which areas within the cortex may be promising candidates for entrainment therapy (Sermon et al., 2023). These evaluations may also identify the pathologies that experience the largest entrainment benefits, increasing the efficiency and impact of the therapeutic application of entrainment moving forward.

Ambiguity across cognitive testing variables further complicates entrainment research. Methodological variation in factors such as stimuli presentation mode and the specific cognitive process studied make it challenging to identify themes across the diverse body of entrainment research. Limited concurrent experimentation prevents researchers from concluding which executive functions or higher-order cognitive processes may reap the largest benefits from entrainment. Reviews on cognitive entrainment alone suggest that attentional processes and memory may be affected more than other cognitive processes (Calderone et al., 2014), but whether this is true for cognitive-motor entrainment paradigms is unknown. Additionally, most cognitive-motor research uses auditory stimulation to evaluate cognitive functioning. However, behavioral and neurobiological psychology tells us that different neural processes are involved in processing visual, auditory, sensory, or kinesthetic information. Visual and kinesthetic stimuli are thought to be easier to remember than auditory stimuli (Bigelow and Poremba, 2014). Perhaps the entrainment of cognitive information is dependent on the sensory modality. Are entrainment benefits more easily identifiable in auditory memory because of the relative challenge of the memory task? Or will larger effects be seen with visual or kinesthetic stimuli presentations?

Individual differences are also likely to influence entrained task performance outcomes. Data from a physio-neuroendocrinological experiment brings forward the idea that individuals with lower cognitive performance abilities at baseline will benefit more from cognitive-motor entrainment (Schmidt-Kassow et al., 2013b). In this study, participants learned 80 Polish-German word pairs and randomly assigned to one of three learning conditions—cycling before learning, cycling during learning, and seated rest before learning. While participants in both physical activity groups remembered more words at a 48-h follow-up test, the sedentary group, individuals with lower verbal memory capacity, performed significantly better in the entrained condition alone. Expanding our understanding of how variation in cognitive functioning ability influences the efficacy of a cognitive-motor entrainment paradigm may help researchers identify which subset of the population may benefit the most, allowing them to target their research efforts and develop therapeutic techniques more efficiently and effectively. Age differences may also impact outcomes. A recent meta-analysis reports that physical activity interventions have a larger beneficial impact in children than adults (Schmid et al., 2023). At the moment, no cognitive-motor entrainment interventions have evaluated cognitive outcomes in a younger population, making this a potential avenue for future research as well.

### 4.2 Important considerations and limitations

Because of the limited amount of research on neural entrainment, it can be challenging to experimentally distinguish between true neural entrainment and fluctuations in rhythmic evoked potentials that directly result from stimuli presentation timing (Haegens and Zion Golumbic, 2018). When an individual can anticipate and predict the presentation of stimuli, their sensitivity to the timed stimuli and their ability to tune out noise increases (Auksztulewicz et al., 2019). This phenomenon has caused some researchers to question if the alignment of neural oscillations causes the cognitive benefits reported in studies on neural entrainment or if it is simply due to the predictability of stimuli presentation (Zoefel et al., 2018). Animal studies and clinical studies on humans with atypical neural functioning point toward variation in the activation of distinct subcortical structures to support either theoretical option (Breska and Ivry, 2018). But, experimental studies on a healthy adult population often result in unclear evidence. Bouwer et al. (2020) attempt to delineate the effects between beat-based and memory-based timing of stimuli presentation in their experiment using EEG and behavioral analyses. Both stimuli presentation conditions lead to cognitive enhancements in an auditory detection task. Concurrent EEG analyses suggest that the mechanisms behind behavioral changes result in similar P1 and N1 attenuation as a response to stimuli presentation. However, the neural benefits from the beat-based entrained condition uniquely decreased the detection of out-of-phase noise, providing evidence to support the theorized patterns of fluctuating attention associated with oscillatory entrainment. Further research is needed to clarify if there is a mechanistic distinction between cognitive benefits thought to arise from neural entrainment and temporally predictable stimuli. Enhancing the scientific community's understanding of the neural activities that influence behavior will help elucidate how to leverage these mechanisms to enhance learning and cognitive functioning.

Additional methodological concerns arise when considering the feasibility of confirming neural entrainment through EEG analysis during a cognitive-motor dual task. Traditional EEG practices ask participants to minimize motor production as any muscular activity producing motor output will increase the likelihood of EEG artifacts, decreasing the quality of data reflecting cognitive activity. But, when using EEG to evaluate the cognitive impact of a physical activity intervention in real-time, the methodological designs needed to test proposed hypotheses often require a participant to engage in movement. Though significant developments have been made in the past decade to increase data quality through different pre-processing techniques (Schmidt-Kassow and Kaiser, 2023), researchers should consider how EEG dual-task data may inherently reflect different neural mechanisms than those from the single-task EEG data collected in more traditional neuropsychological experiments.

Most importantly, challenges arise when considering how laboratory data may be translated to a real-world setting so that individuals outside the academic community can realize potential benefits from a cognitive-motor dual-task. While initial studies in controlled environments report cognitive benefits from cognitive-motor entrainment, it is possible that environmental variation could alter the production of motor output and influence anticipated effects. As most entrainment research aims to enhance our understanding of neural entrainment for its beneficial use, the methodological designs of experiments should cater to real-world applications. Cognitive-motor entrainment designs incorporating simple body-weight physical activities are likely to be integrated into a community-based intervention most easily. Considerations should also be made to ensure participants engage in age-appropriate cognitive-motor tasks to increase the likelihood of an intervention's success.

## 5 Conclusion

Cognitive-motor entrainment research draws from an interdisciplinary network of knowledge stemming from work in psychology, neurology, kinesiology, etc. Under the appropriate conditions, neural entrainment decreases cognitive effort and attentional demands, enhancing cognitive and motor functioning. This principle can be extended and applied to tasks that require synchronous cognitive and motor output. Research suggests that cognitive-motor entrainment alleviates dual-task costs and enhances cognitive functioning. Further exploration of this principle may allow researchers and practitioners to utilize its benefits and maximize positive outcomes following rehabilitation or learning interventions. For example, this principle may be applied to therapeutic techniques seeking to alleviate the loss of motor skills following a cerebral vascular accident, a classroom-based setting to augment the potential for learning and increase physical activity levels among students, or possibly used as a diagnostic tool to evaluate an individual's ability to manage different levels of cognitive load during task completion. By prioritizing research that seeks to understand how to maximize the benefits of cognitive-motor entrainment, scientists will be able to test its theoretical foundations and determine if this principle holds promise moving forward.

## Author contributions

DS: Writing – original draft, Writing – review & editing.
